# Case report of metastatic invasive breast lobular carcinoma to the urinary bladder

**Published:** 2016-01-01

**Authors:** Ahmed A. Al Ibraheemi

**Affiliations:** Resident in Clinical Oncology, Al Basheer Hospital, Amman, Jordan

**Keywords:** Breast cancer, Metastasis, Urinary bladder

## Abstract

Breast cancer is the most common cancer in women except skin cancer. The common metastatic sites include lymph node, lung, liver and bone. However, metastasis to the bladder is extremely rare. To our knowledge, this is the first case of breast cancer metastasis to urinary bladder in Jordan which is reported. Nine years after the initial diagnosis of lobular breast carcinoma, the patient suffered from left side leg edema; Ultrasonography and Computed tomography scanning showed thickening of posterior bladder wall and bilateral hydronephrosis. The biopsy of the bladder confirmed metastatic lesion from the breast. In contrast to the primary tumor, bladder metastasis showed negative expression of estrogen (ER) and progesterone (PR) receptors. However, Her2neu test was negative in both. The reported case confirms that bladder metastasis from breast cancer tend to occur late after the diagnosis of the primary tumor. Furthermore, bladder metastasis can be asymptomatic and heterogeneous in ER and PR expression in comparison with the primary tumor. This report supports the need for careful follow-up and early intervention whenever such clinical situation is suspected. This report supports further evaluation of receptor status at time of metastasis.

## Introduction

 Breast cancer is the second most common cancer in the world and by far, the most frequent cancer among women with an estimated 1.67 million new cancer case diagnosed in 2012 (25% of all cancers).^1^ Compared with developed countries (794,000), more cases were reported in under-developed countries (883,000 cases). Incidence rates vary nearly four-fold across the world regions, with rates ranging from 27 per 100,000 in Middle Africa and Eastern Asia to 96 in Western Europe.^1^ Malignant breast tumor metastasize to lungs, bone, liver, lymph nodes and skin; less frequently it can also spread to brain, adrenal glands, ovaries, spleen, pancreas, kidneys, thyroid and heart.^2^ Metastasis to urinary bladder from solid tumors is rare and represents 2% of all bladder neoplasms.^3^ Direct extension from the cervix, prostate and colon is not unusual but metastasis from a distant organ is extremely rare. Breast cancer represents as a primary site in about 2.5% cases of all metastatic bladder cancer.^3^ We reported case of metastatic invasive breast lobular carcinoma to the urinary bladder.

## CASE REPORT

 Our patient is a 50 year old female diagnosed with breast cancer. She had negative medical and family history regarding breast cancer. Her first menstrual history was at age 14; she became a pregnant at 24 years old. The patient has 6 children all of them were breast fed, each child fed for 1 – 1.5 year.

Early 2005, she presented with painless right breast mass in upper outer quadrant with nipple and skin retraction. This had been going for about 3 months. 

Mammogram showed a large high dense soft mass lesion in the upper outer quadrant of the right breast with speculated outline measured (26 X 30 mm). Fine-needle aspiration showed invasive carcinoma. Subsequently, quadrectomy with ipsilateral axillary lymph node dissection was done; tumor size was (5 X 4.5 cm) with no skin involvement. Histopathological examination revealed (invasive lobular carcinoma) with some elements of (lobular carcinoma in situ), with three out of four lymph nodes were involved. Tumor stage was pT2pN1M0. ER and PR receptors were positive and Her2neu was negative. Chest, abdomen and pelvis CT scan and bone scan were normal.

After 2 months she started chemotherapy (FAC X 6) protocol, (5 FU 800 mg) (ADM 80 mg) (cyclophosphamide 800 mg), then she received radiotherapy (42.9Gy / 13 Fx) to the right chest wall and lymphatic area and then (10 Gy / 5 Fx) to the right posterior axillary area. Hormonal therapy was started in July 2005 (Tamoxfin Tab. 20 mg once daily) then in the beginning of 2009 she was switched to (Arimidex Tab 1 mg once daily).

**Figure 1:**
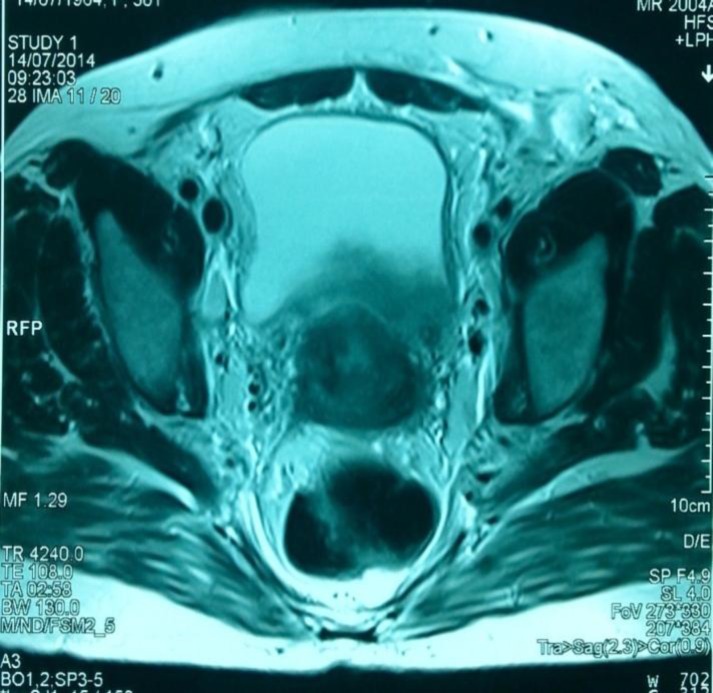
50-year-old female patient with invasive lobular breast carcinoma metastasized to the urinary bladder. Contrast-enhanced MRI image of the pelvis with axial reconstruction demonstrating hyper-dense segmental urinary bladder wall thickening involving posterior wall of the bladder

**Figure 2:**
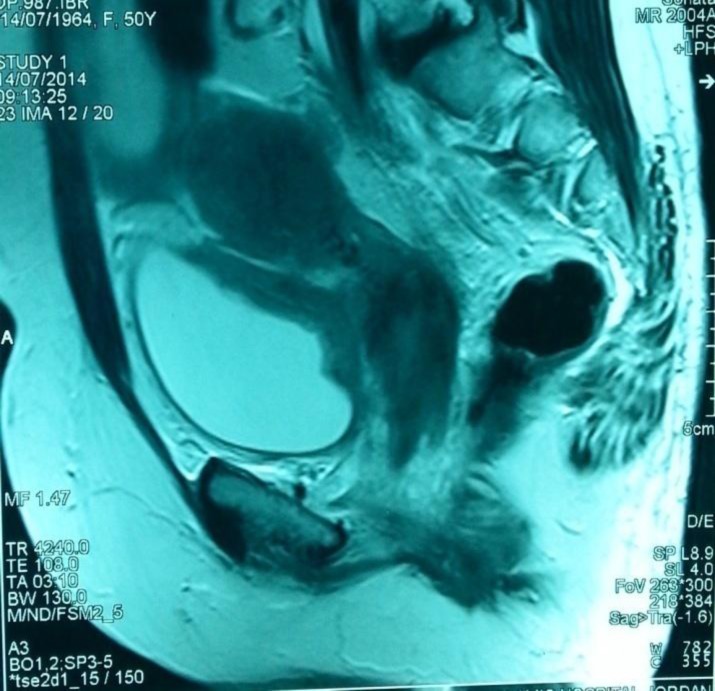
50–year-old female patient with invasive lobular breast carcinoma metastatic to the urinary bladder. Contrast Enhanced MRI image of the pelvis, sagittal reconstruction demonstrating: thickening of posterior urinary bladder wall

**Figure 3:**
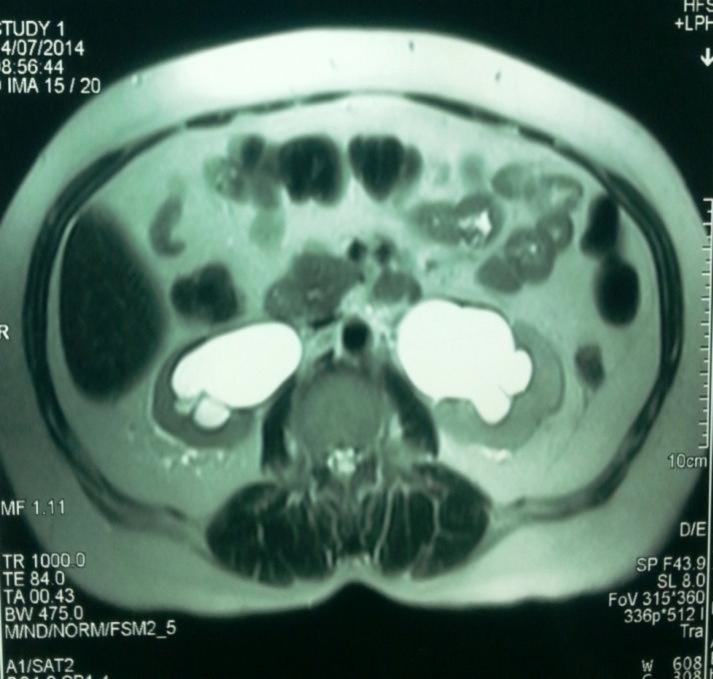
50-year-old female patient with invasive lobular breast carcinoma metastatic to the urinary bladder. Contrast Enhanced MRI image of the pelvis, axial reconstruction demonstrating: bilateral hydronephrosis

She was on regular follow up every 6 months till July 2014, when the patient suffered from left leg pitting edema. Blood urea and serum creatinine were (14.9 mmol/ L) and (185 Umol/L), respectively. Abdomino-pelvic ultrasonography had shown moderate hydronephrosis seen in bilateral pelvi-calyceal system with hydroureter; also, it had shown thickening in posterior wall of the urinary bladder. Pelvic CT scan and MRI confirm ultrasonography results and shown irregular thickening of bladder posterior wall (Figure 1, 2, 3), bilateral iliac and inguinal lymph node enlargement which noticed more on left side, bilateral pleural effusion (moderate on left and mild on right side). Multiple metastases were seen in dorsal and lumbar vertebrae in addition to pelvic bones metastasis.

The urologist inserted bilateral double J stent in both ureters. Cystoscopy showed thick irregular posterior bladder wall with no definitive mass lesion; so, random bladder wall biopsies were taken and it revealed infiltration of bladder mucosa by nests and single tumor cells, these cells have plasmocytoid appearance and morphologically simulating metastatic breast carcinoma. The results of Immunohistochemistry study showed positive CK7, negative CK20 and positive CD 138. These findings favor the diagnosis of metastatic breast carcinoma (mostly lobular subtype); hormonal analyses were ER, PR and Her2neu negative.

## Discussion

 Primary malignancy of the bladder is the fourth and ninth most common cancer in males and females, respectively. Nonetheless, the bladder is only rarely a metastatic site of other cancers and, as a consequence, is seldom evaluated in the clinical follow-up of patients affected by malignant neoplasia.^4^

The presenting symptom of our case was left leg pitting edema. Clinical presentation may vary, ranging from the most common painless hematuria, stress and urge incontinence, frequency and nocturia to rare signs like dysuria and back pain.^5^

Few cases of bladder metastasis from breast cancer leading to symptomatic hydronephrosis, renal failure, and death are described.^6^^,^^7^ However, asymptomatic cases had been reported.^8^ In the majority of reported cases, the urinary bladder lesions from breast cancer were part of systemic dissemination. This observation indicates that bladder metastases are typically late complications of primary disease.^9^

In our reported case, in addition to bladder metastasis, bone metastasis was seen in Abdomino-pelvic CT scan and MRI. The metastatic patterns of lobular and ductal carcinoma of the breast are different, with gastrointestinal system, gynecological organ, and peritoneal-retroperitoneal metastases markedly more prevalent in lobular carcinoma.^10^ Bladder metastasis in the present case was part of systemic metastasis seen in bone and pleural effusion that confirmed by CT scan and MRI. The above findings are compatible with Zagha et al. study which demonstrated that, in the majority of reported cases, the urinary bladder lesions from breast cancer were part of systemic dissemination.

This observation indicates that bladder metastases are typically late complications of primary disease.^9^

The metastatic pattern of breast carcinoma may be related to the histologic type of cancer.^7^ In our case, the primary and metastatic histopathology was invasive lobular carcinoma; this type of histopathology (which according to multiple sources accounts for 8-14% of all cases of breast primary tumors), has a higher bladder metastatic rate in comparison with infiltrating ductal carcinoma (which representing 65-85% of breast cancers). It is postulated that breast neoplasm metastasizes to the bladder via retroperitoneal lymphatic involvement, observed more frequently in ILC cases.^11^ In which, metastatic spread was seen more frequently in the bones and abdominal region whereas IDC metastasized to the lung. There was no difference in liver, nonaxillary lymph nodes or the central nervous system,^12^ the difference could be due to a cell size or shape with physical properties that favor certain areas with microanatomy that is more conducive to stopping or trapping these types of cells. Alternatively, the microenvironment of the ovary or peritoneum may provide growth and survival factors that favor ILC cells over IDC cells. Additional molecular or biologic differences might account for this peculiar pattern of metastasis. It has been demonstrated that loss of expression of the cell–cell adhesion molecule E-cadherin in ILC may decrease adhesiveness of cells and facilitate this type of infiltration.^13^

Immunochemistry staining for various markers is routinely used in diagnosis of undifferentiated tumor. Cytokeratin, CK-7, CK-18, CK-19, CK-20 are useful screening markers for the recognition of epithelial differentiation.^14^

In the present case, positive (CK7 and CD 138) and negative CK20 helped the pathologist in confirming the diagnosis of metastatic breast cancer. Additionally, we noticed that there were differences in hormonal expression between primary and metastatic tissues; while primary tumor was ER, PR positive and Her2neu negative, metastatic tumor was negative for ER, PR and Her2neu. The present findings are in line with previous studies which showed discrepancy between receptors is not uncommon between the primary and the secondary tumor (reported between 30% and 39%).^7^^,^^10^^,^^15^ The hypotheses of the heterogeneous ER and PR expression in the primary tumor and metastatic lesions in the same patient include: the breast cancer cells are polyclonal; and ER and PR expression may change after endocrine therapy, due to the elimination and growth of ER- and PR positive or negative cells or due to gene mutations.^15^

## CONCLUSION

 To our knowledge, this is the first case report of metastatic breast cancer to urinary bladder registered in Jordan. High level of awareness needed regarding metastatic sites that may be invaded by primary breast cancer especially (lobular subtype). Besides, appearance of urinary symptoms is not necessary indicative of bladder metastasis. Therefore, close follow up of patient by frequent ultrasound is mandatory for early detection of metastasis.
